# Functions of Representative Terpenoids and Their Biosynthesis Mechanisms in Medicinal Plants

**DOI:** 10.3390/biom13121725

**Published:** 2023-11-30

**Authors:** Qingjie Wang, Xiya Zhao, Yang Jiang, Biao Jin, Li Wang

**Affiliations:** College of Horticulture and Landscape Architecture, Yangzhou University, Yangzhou 225009, China; wangqingjie@yzu.edu.cn (Q.W.); mz120221406@stu.yzu.edu.cn (X.Z.); mz120201267@stu.yzu.edu.cn (Y.J.); bjin@yzu.edu.cn (B.J.)

**Keywords:** terpenoids, medicinal plants, function, molecular regulation mechanisms

## Abstract

Terpenoids are the broadest and richest group of chemicals obtained from plants. These plant-derived terpenoids have been extensively utilized in various industries, including food and pharmaceuticals. Several specific terpenoids have been identified and isolated from medicinal plants, emphasizing the diversity of biosynthesis and specific functionality of terpenoids. With advances in the technology of sequencing, the genomes of certain important medicinal plants have been assembled. This has improved our knowledge of the biosynthesis and regulatory molecular functions of terpenoids with medicinal functions. In this review, we introduce several notable medicinal plants that produce distinct terpenoids (e.g., *Cannabis sativa*, *Artemisia annua*, *Salvia miltiorrhiza*, *Ginkgo biloba*, and *Taxus media*). We summarize the specialized roles of these terpenoids in plant-environment interactions as well as their significance in the pharmaceutical and food industries. Additionally, we highlight recent findings in the fields of molecular regulation mechanisms involved in these distinct terpenoids biosynthesis, and propose future opportunities in terpenoid research, including biology seeding, and genetic engineering in medicinal plants.

## 1. Introduction

Medicinal plants are defined by the World Health Organization as plants that contain a substance in one or more organs that can be used for therapeutic purposes or as a precursor for the synthesis of a useful drug [1]. The biologically active constituents in medicinal plants are mainly terpenoids, alkaloids, and phenylpropane compounds [2]. Terpenoids, in particular, constitute a significant and varied group of natural products that includes both non-volatile and volatile compounds. Currently, over 80,000 terpenoid compounds have been identified and are widely used in the pharmaceutical and cosmetic industries [3,4].

**Figure 1:**
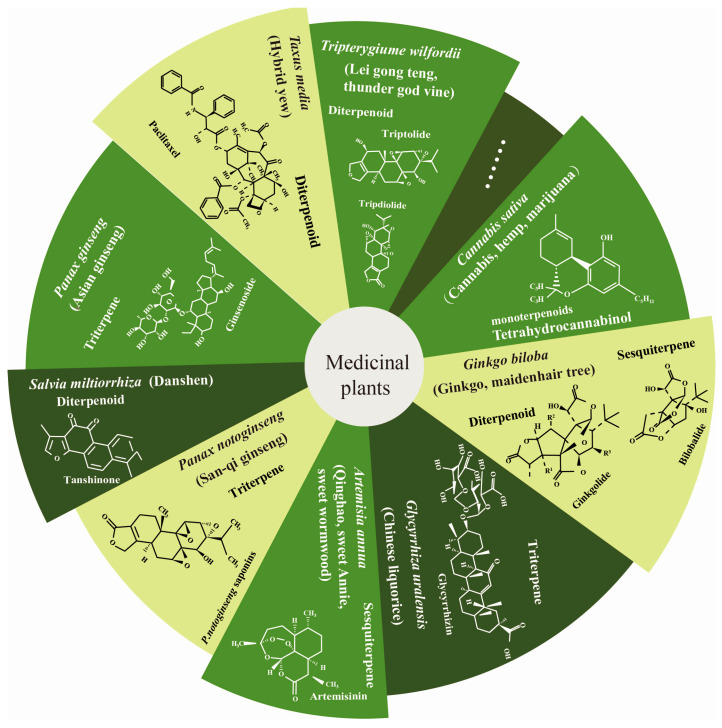
Several important medicinal plants and their specific terpenoids.

All terpenoids comprise five-carbon isoprene units (C_5_H_8_). Based on the number of these units, they can be classified as monoterpenes, sesquiterpenes, diterpenes, sesterterpenes, triterpenes, tetraterpenes, and polyterpenes [5,6]. Some specific terpenoids with unique medicinal properties have been identified in certain medicinal plants (Figure 1). These include cannabinoids (a constituent of *Cannabis sativa*), tanshinone (a constituent of *Salvia miltiorrhiza*), paclitaxel (a diterpenoid compound isolated from *Taxus media*), artemisinin (a bioactive compound of *Artemisia annua*), ginsenoside (a triterpene compound from *Panax ginseng*), and ginkgolide (a component of *Ginkgo biloba*) [7,8,9,10,11]. These terpenoids not only play a significant role in the management of cardiovascular and cerebrovascular diseases and malaria but can also improve the resistance of plants to biotic and abiotic stress [12]. In turn, certain stressors and their associated hormones can promote the accumulation of terpenoids, demonstrating a feedback mechanism in terpene biosynthesis. In addition, advances in high-throughput sequencing technology have led to the successful assembly of more than 195 medicinal plant genomes and the discovery of the synthetic metabolic pathways of essential terpenoids [13,14]. The terpenoids found in medicinal plants are numerous and serve a wide variety of medicinal functions. In this regard, we have identified several major terpenoids that are well-known for their benefits in medicine. These include pain-relieving cannabinoids, anti-malarial artemisinins, anti-cardiovascular tanshinones and ginkgolides, and anti-tumor paclitaxel. Extensive research has been devoted to studying these terpenoids due to their significant contributions to human health and their potential in drug discovery. These terpenoids are among the most potent and promising compounds in the field of medical applications. Our paper provides a summary of the types and functions of terpenoids in the main medicinal plants. We specifically focus on the mechanisms of structural genes, transcription factors, and transporters participating in the regulation of terpenoid biosynthesis. Additionally, we discuss and compare the terpenoid biosynthesis pathway in these medicinal plants. Given that medicinal plant terpenoids are numerous and specific, we concentrate on the terpenoids of several representative medicinal plants, as defined in Figure 1.

## 2. Function of Terpenoids in Human Health

Recent studies have demonstrated that terpenoids in medicinal plants possess pharmacological properties that promote human health [15,16]. For example, artemisinin is a widely used anti-malarial medicine that is used to protect millions of people from malaria every year [17,18]. Ginkgolides are natural antagonists of platelet activation, possess neuroprotective and reparative effects, and are crucial in the treatment of ischemic strokes [19]. Cannabinoids have been used for thousands of years for their anxiolytic and anesthetic effects [20]. Paclitaxel plays a significant role in anti-tumor therapy by inducing oxidative stress, and is considered one of the most successful natural anti-cancer drugs available [21]. Furthermore, terpenoids have been found to have additional biological functions, such as anti-oxidant and anti-inflammatory effects [22,23].

Terpenoids exert their therapeutic functions through various signaling pathways and receptors. For example, cannabinoids rely on their ability to interact with the body’s endocannabinoid system—the G protein-coupled cannabinoid receptors (CB1 and CB2)—to exert their biological properties, including infection, inflammation, and hard tumors [24]. Due to the complex and diverse involvement mechanisms of terpenoids, we mainly focused on their signaling pathways in inflammatory and oxidative stress. NF-κB (Nuclear factor-kappa B) is involved in inflammatory processes, cell proliferation, and defense against apoptosis [25,26]. Artemisinin has been shown to significantly inhibit the expression of NF-κB and mitogen-activated protein kinase (MAPK) signaling genes, making it a potential drug for inflammation treatment [27]. Tanshinone IIA has been reported to play a role in the inflammatory response through the Toll-like receptor 4 (TLR4)/transforming growth factor activated kinase-1 (TAK1)/NF-κB signaling cascade [28,29,30]. Cannabinoids regulate anti-inflammatory responses by inhibiting NF-κB and other pathways [31]. Ginkgolides can significantly inhibit the expression of NF-κB, reduce the phosphorylation levels of p38, c-Jun N-terminal kinase (JNK), and extracellular signal-regulated kinase (ERK) proteins in the mitogen-activated protein kinase (MAPK) signaling pathway, and reduce the expression of pro-inflammatory cytokine genes, thereby exerting anti-inflammatory effects [32]. Similarly, paclitaxel can inhibit the expression of NF-κB, inhibiting tumor growth and having anti-tumor effects [33].

Oxidative stress plays a crucial role in antitumor activity, cardiovascular activity, and atherogenesis [33,34,35,36]. Ginkgolide can cross the blood–brain barrier, activating the nuclear factor E2-associated factor 2 (Nrf2) signaling pathway and upregulating the expression of oxidative stress-related proteins heme oxygenase-1 (HO-1), quinone oxidoreductase 1 (NQO1), superoxide dismutase (SOD), and Nuclear factor-E2-related factor 2 (Nrf2) to reduce the cellular damage caused by oxidative stress [31]. Oxidative stress is central to the mechanism of paclitaxel’s antitumor activity as it increases mitochondrial ROS production and inhibits endoplasmic reticulum utilization and antioxidant enzymes and peptides [33,34,35,37,38]. Recent studies have found that tanshinone IIA prevents oxidative stress by increasing the activities of total antioxidant capacity (T-AOC), SOD, glutathione peroxidase (GSH-Px), and catalase (CAT) to reduce atherosclerosis [35,39]. Cannabinoids regulate antioxidants by activating antioxidant enzymes, regulating glutathione levels, and inhibiting pro-oxidases to regulate anti-inflammatory properties [40]. Although it has been discovered that terpenoids can be involved in treating diseases through multiple strategies, the specific regulatory pathways for disease treatment are not yet fully understood. Further validation is still required via utilizing more animal models or clinical trials to elucidate their regulatory mechanisms.

## 3. Functions of Terpenoids in Biotic Stress

On an evolutionary level, it seems that there are complex and diverse combinations of terpenoids with remarkable structural diversity that can fulfill a variety of ecological roles [41]. The terpenoids in medicinal plants also serve an ecological role in defending the plants themselves against herbivorous insects and pathogens. Terpenoids can attract natural enemies of aphids, such as predators and parasitoids. Increasing the cannabinoid content in *C*. *sativa* leaves can improve resistance to mites, especially *T*. *urticae* [42]. In *A*. *annua*, a high level of artemisinin increases the resistance of *Pseudomonas syringae*, while a low level reduces resistance to *Botrytis cinerea* [43]. When attacked by aphids, *A*. *annua* also produces artemisia ketone and (E)-β-farnesene to resist them [44]. These results suggest that biotic stress can induce *A. annua* to produce more and diverse types of artemisinin to enhance the plant’s resistance. Terpenoids not only protect plants themselves, but also act as alarm signals at interspecific, intraspecific, and intraplant levels. These signals trigger defense responses in nearby plants or tissues that have not yet been attacked [45]. Therefore, due to their low environmental risk and resistance to pests and pathogens, terpenoids can be effectively used as biopesticides, playing a vital role in environmental protection.

## 4. Effects of Environmental Factors on Terpenoid Accumulation

**Figure 2:**
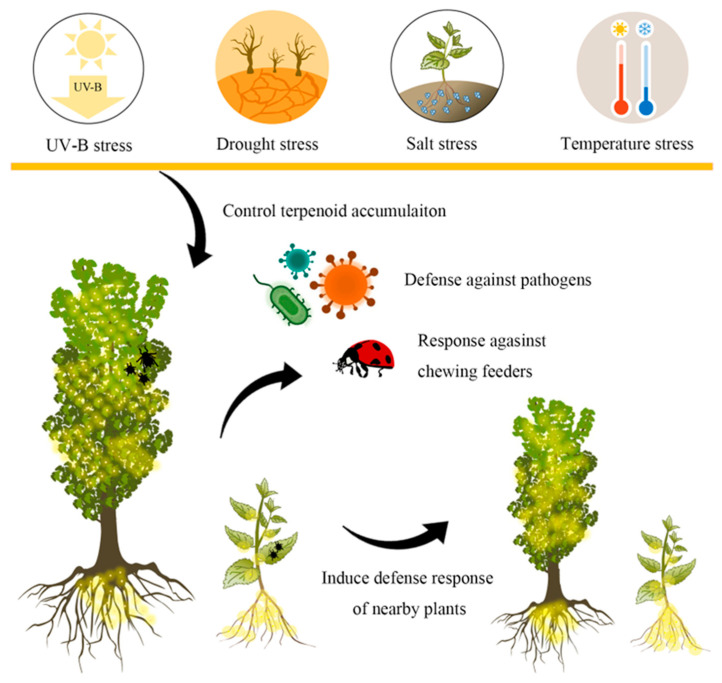
Terpenoid-mediated plant-environment interactions. Medicinal plants produce terpenoids to inhibit insect feeding and pathogen attack, whereas UV-B radiation, drought, salt, and temperature stress can induce terpenoids accumulation in medicinal plants.

Synthesized and accumulated terpenoids are tightly regulated by changing abiotic environments (Figure 2) [46,47]. Light plays a critical role in terpenoid accumulation, which cannabinoid synthesis is a wonderful example. To achieve a consistent production of cannabis products, it is increasingly being grown indoors using Light Emitting Diode (LED) light, which is more energy efficient than traditional high-pressure sodium (HPS) light sources. Higher cannabinoid concentrations are measured under LED lighting compared to HPS [48]. Similarly, cannabigerolic acid (CBGA) was accumulated up to 400% more during LED treatments compared to HPS [49]. Moreover, combining LED light and metal halide lamps increased bud yield and cannabinoid concentrations [50]. Blue light LED can also lead to increased cannabinoid concentration in *C*. *sativa* [51,52,53]. The dynamic spectrum LED treatment leads to increase in yield and significant improvement in cannabinoid content compared to fixed full-spectrum white light [54].When plants are exposed to a strong light intensity or ultraviolet-B (UV-B) radiation, they can initiate the terpenoid metabolite biosynthesis for antioxidant defense [55]. For instance, there is a positive association between UV-B radiation and cannabinoid content in *C*. *sativa* [56]. After exposure to UV light for 24 h and 48 h, the terpenoid content increased by 10.0% to 21.9% in *G. biloba* [57]. Artemisinin accumulation also correlates positively with light intensity, with the highest concentration observed at 3000 Lux when exposed to a standard fluorescent lamp [58]. 

Cold stress, such as chilling (0–15 °C) and freezing (<0 °C), adversely affects plant development, but facilitates terpenoid accumulation [59]. The artemisinin content increased by 27.16% upon exposure to cold stress and overnight frost in *A*. *annua* [60,61]. In addition, cold stress (4 °C) also significantly increased the terpenoid content in *G. biloba*, reaching a maximum at 8 d and increasing by 14.5% compared to the control [62]. 

Salt stress can increase the content of artemisinin by 14.68%, but prolonged salt stress decreases artemisinin accumulation [63]. In *C*. *sativa*, an increase in NaCl concentration was also found to result in a subsequent decrease in cannabinoid content [64,65]. Drought can enhance terpenoid accumulation in medicinal plants (e.g., sesquiterpene content of *Salvia dolomitica*) [66]. The emission rates of terpenoid compounds are higher in cork oak and gum rock with increasing summer drought [67]. Similarly, there was a significant increase in the amount of (E)-β-caryophyllene in *O*. *vulgare* subsp. virens due to the drought [68]. Controlled drought stress can boost cannabinoid content in *C*. *sativa* [69]. Further research has shown that under drought stress, tanshinone enhances the photosynthetic capacity and prevents oxidative damage, thus improving drought stress resistance in *S*. *miltiorrhiza* [70].

Although the existing studies have discovered various external factors that can regulate terpenoid synthesis in medicinal plants, research on the regulatory pathways affected by these environmental factors is still very limited. Due to the importance of the environment in promoting the accumulation of terpenoids, further research should be conducted in the future to investigate various environmental factors and their combinations that regulate terpenoids accumulation in medicinal plants. By considering the characteristics of different medicinal plants, the most advantageous combination of environmental factors for terpenoid accumulation should be optimized and selected. This will provide valuable insights for the future development of high-quality medicinal plants through factory cultivation.

## 5. Accumulation Characteristics of Terpenoids and Their Transport

The specific sites of terpenoid synthesis, storage, and utilization vary among medicinal plants. Multicellular glandular trichomes, which are outgrowths on the epidermal, possess the ability to secrete or store significant amounts of terpenoids. Glandular secretory trichomes, located on the surface of the leaves, are where artemisinin is synthesized and stored. Thus, the density of glandular trichomes affects artemisinin accumulation [71]. Cannabinoids are present in all *C*. *sativa* aerial plants and the resin is the most highly concentrated. Cannabinoids were originally synthesized in the trichoid in the form of cannabic acid. Since acidic substances are highly toxic to plant cells, the final stage of their synthesis takes place outside the cells of the trichosomes [72]. The roots of *C*. *sativa* also contain triterpenoids’ active compounds [73].The high concentrations of monoterpenoids occur in capitate glandular trichomes in bracts; cannabinoids are also stored in the glandular trichome secretory cavity, but they are found predominantly in female flowers [24]. In *T*. *media*, taxol exhibits special mechanisms for synthesis and storage to prevent toxic effects on its source plant. Taxol can be isolated from nearly all its plant tissues, but its content is higher in the stems, primarily accumulating in the outer bark, compared to the roots and needles [74]. The present studies also reveal that taxol is stored in hydrophobic bodies within the wood parenchyma rays and phloem. This storage can help protect living cells from the cytotoxic effects of taxol [75]. By contrast, in *G*. *biloba*, the terpenoids are primarily extracted from the leaves. However, experiments using ^14^CO_2_ and (U-^14^C) glucose-labeled revealed that their biosynthesis site is not in the leaves but in the roots [76]. The content of terpenoids found within the cortex (bark) was 1.75 to 2.07 times higher than that found in the leaves. *GbLPS*, *GbIDS2*, *GbDXR2*, *GbDXS2*, and *GbGGPPS*, the core genes in the ginkgolide biosynthetic pathway, exhibited a high level of expression in roots, confirming that the roots are the main site of terpenoid biosynthesis [77,78]. Metabolic profiling and gene expression analysis indicate that ginkgolides are biosynthesized in the root fibrous and main root periderm tissues, and subsequently transported to the leaves [78]. Similarly, several major bioactive compounds have been extracted from the roots of *S*. *miltiorrhiza*, including cryptotanshinone and tanshinone IIA. The genes relevant to transhinone synthesis, particularly *SmKSL1*, *SmCPS1*, and 19 candidate *SmCYPs*, are all highly expressed in root periderm tissue [79,80], confirming that tanshinone accumulation mainly occurs in the *S*. *miltiorrhiza* root phloem. 

Since some terpenoids are produced and stored at different sites in plants, it is necessary to study their transport [81]. However, the transport mechanisms of terpenoids have not been extensively researched in medicinal plants. ATP-binding cassette (ABC) transporters are present in most eukaryotic organisms, and can be categorized into eight subfamilies (ABCA to ABCH). They can drive the influx or efflux of secondary metabolites into cells [82,83]. A few transporters from the ABCG subfamily are implicated in terpenoid transport. The pleiotropic drug resistance (PDR) transporters, belonging to the ABCG subfamily, were found to be responsible for the membrane transport of terpenoids in medicinal plants [84,85]. In *P*. *ginseng*, *PgPDR3* has been implicated in corresponding ginsenoside accumulation [85]. Similarly, in *A*. *annua*, *AaPDR3* is involved in both the formation and transport of β-caryophyllene [86]. Additionally, the genome of *S*. *miltiorrhiza* contains a total of 114 genes that encode ABC transporters [83]. Combined with analyzing their expression in different tissues and their co-expression with key enzymes involved in terpenoid synthesis, several ABCG transporter genes, such as *SmABCG46*, *SmABCG40*, and *SmABCG4*, have been identified as potentially participating in tanshinone transport [83]. Although numerous terpenoid transporters have been identified in medicinal plants, validating their functions and determining their specificity for substrates remains challenging.

## 6. Terpenoid Biosynthesis Pathway and Regulation

**Figure 3:**
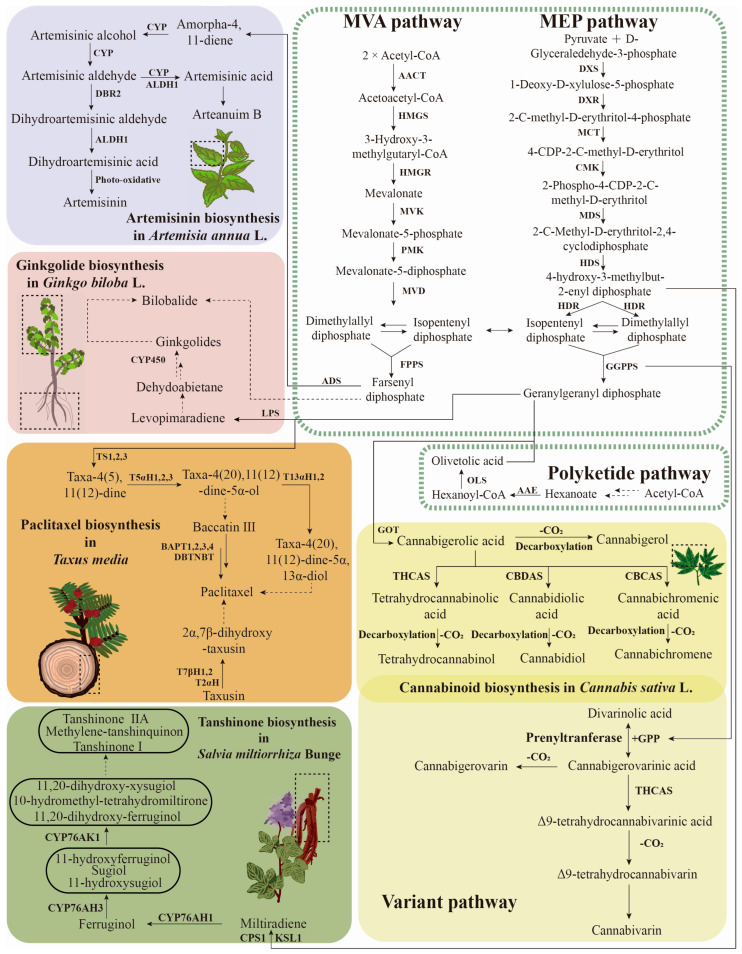
Biosynthetic pathways of terpenoids in several important medicinal plants. Artemisinin (purple), cannabinoids (orange), ginkgolide (pink), paclitaxel (yellow), tanshinones (green). One solid arrow indicates one step, two solid arrows represent more than one step, and the dashed arrows show hypothetical steps. Abbreviations: AACT, acetoacetyl-CoA synthase; HMGR, HMG-CoA reductase; HMGS, hydroxymethylglutaryl (HMG)-CoA synthase; MVK, mevalonate kinase; PMK, phosphomevalonate kinase; MVD, mevalonate diphosphate decarboxylase; FPPS, farnesyl diphosphate synthase; DXR, 1-deoxy-D-xylulose-5-phosphate reductase; DXS, 1-deoxy-D-xylulose-5-phosphate synthase; HDR, 1-hydroxy-2-methyl-2-(E)-butenyl-4-diphosphate reductase; HDS, 1-hydroxy-2-methyl-2-(E)-butenyl-4-diphosphate synthase; CMK, 4-diphosphocytidyl-2-C-methyl-D-erythrito kinase; MCT, 4-diphosphocytidyl-2-C-methyl-D-erythrito synthase; MDS, 2-C-methyl-D-erythritol-2, 4-cyclodiphosphate synthase; GGPPS, geranylgeranyl diphosphate synthase; ADS, amorpha-4, 11-diene synthase; ALDH1, aldehyde dehydrogenase 1; DBR2, artemisinic aldehyde Δ11(13) reductase; LPS, levopimaradiene synthase gene; CYP450, cytochrome P450; TS, taxadiene synthetase; T5αH, taxadiene 5-α-hydroxylase; T2αH, taxoid 2-α-hydroxylase; T7βH, taxoid 7-β-hydroxylase; TBT, 2-α-hydroxytaxane 2-O-benzoyltransferase; BAPT, baccatin III amino phenylpropanoyl-13-O-transferase; DBTNBT, 3′-N-debenzoyltaxol-N-benzoyltransferase; T13αH, taxane 13-α-hydroxylase; KSL, kaurene synthase-like; CPS, copalyldiphosphate synthase; AAE, acyl-activating enzyme; OLS, olivetol synthase; GOT, olivetolate geranyltransferase; THCAS, tetrahydrocannabinolic acid synthase; CBDAS, cannabidiolic acid synthase; CBCA, cannabichromenic acid synthase. Dashed square frames represent terpenoid biosynthesis sites.

Despite their substantial differences in structure and functionality, the terpenoids all originate from isopentenyl diphosphate (IPP) and dimethylallyl diphosphate (DMAPP). These IPP and DMAPP units can be biosynthesized via the mevalonate (MVA) or the methylerythritol phosphate (MEP) pathway [87,88,89]. However, the downstream genes integrated into the terpenoid biosynthetic pathways, and the regulatory mechanisms, are specific to different medicinal plants (Figure 3).

Gene tandem duplication is a prominent feature in the biosynthesis pathways of key secondary metabolites. Two strains of *A*. *annua* with varying levels of artemisinin were used to assemble chromosome-level haploid maps. Genomic analyses revealed that multiple copies of the *AaADS* (amorpha-4, 11-diene synthase) gene exist in the *A*. *annua* genome. A strong positive association was found between artemisinin levels and the number of copies of *AaADS* [71]. ADS catalyzes the initial and critical step in the artemisinin biosynthetic pathway, the conversion of farnesyl diphosphate (FDP) to amorpha-4, 11-diene [90]. Overexpression of *AaADS* can enhance artemisinin production in transgenic *A. annua* plants, suggesting the important role of *AaADS* in artemisinin biosynthesis [91,92]. Then, the cytochrome P450 enzyme CYP71AV1, along with the cytochrome P450 reductase CPR1 and cytochrome b5 (CYB5), effectively converts amorpha-4, 11-diene into artemisinic alcohol71 [93]. The overexpression of *AaCYP71AV1* and *AaCPR* (redox partner for *CYP71AV1*) in *A*. *annua* can increase the artemisinin content by 2.4-fold in comparison to the wild-type [94,95]. Several other genes which are important for artemisinin synthesis have also been identified, including *AaHMGR*, *AaDBR2*, *AaDXR*, and *AaFPS* [96,97]. The co-transformation of the two important synthetic genes (e.g., *AaADS* and *AaHMGR* genes, *AaFPS* and *AaHMGR* genes) can significantly promote artemisinin content [96]. In particular, the co-overexpression of *AaFPS*, *AaCYP71AV1*, and *AaCPR* can dramatically increase the artemisinin concentration in *A*. *annua* [98]. In addition, most of these genes can be activated with UV light, cold, or heat shock, which correlates with higher artemisinin content [43,99,100,101,102,103].

In *S*. *miltiorrhiza*, tanshinone biosynthesis is initiated by the bicyclization of geranylgeranyl diphosphate (GGPP), a common diterpene precursor, and catalyzed by CPP synthases (CPSs), resulting in copalyl diphosphate (CPP). The subsequent cyclization or rearrangement reaction of the alkene is catalyzed by kaurene synthase-like enzymes (KSLs) to produce an olefin. The final production of diterpenoids requires the insertion of oxygen from cytochrome P450 mono-oxygenases (CYPs) [104,105]. In the genome of *S*. *miltiorrhiza*, 82 terpene synthase genes, which are responsible for producing hemi-, mono-, sesqui-, or di-terpenes, were identified [106]. Physical clustering of terpene synthase genes and CYPs is often linked to consecutive enzymatic actions in the biosynthesis of terpenoids. Four terpenoid synthase genes/CYP pairs have been found in the *S*. *miltiorrhiza* genome. Among these, *SmCPS1* and *SmCPS2* are, respectively, responsible for the tanshinone biosynthesis in roots and leaves, and both are accompanied by genes from the *CYP76AH* subfamily. It is worth highlighting that one of these members, *CYP76AH1* and *CYP76AH3*, was previously identified as playing an important role in tanshinone biosynthesis, confirming the functional role of these biosynthetic gene clusters in the biosynthesis of tanshinone [107]. In addition, biochemical and genetic experiments have also demonstrated the crucial role of several *CYP450* genes implicated in tanshinone biosynthesis, including *SmCYP76AH1*, *SmCYP76AH3*, and *SmCYP76AK1* [108,109,110]. 

In *Taxus* species, the biosynthesis pathway of toxoids is very complex and involves approximately 19 enzymatic steps. The process begins with the precursor GGPP and includes several key enzymes such as a taxadiene synthase (TS), five P450s (T2aOH, T5aOH, T13aOH, T7bOH and T10bOH), five acyltransferases (BAPT, DBAT, DBTNBT, TAT and TBT), and two extra enzymes (PAM and T2′αOH) [111,112]. The TS catalyst facilitates the cyclization of GGPP to form taxadiene. The expression of the TS gene through heterologous transformation in tobacco can significantly increase taxadiene production [113]. The enzymes taxoid 2α-hydroxylase and taxoid 7β-hydroxylase utilize taxusin as a substrate to, respectively, produce 2α-hydroxytaxusin and 7β-hydroxytaxusin [114]. Overexpression of the gene encoding *DBTNBT* increased paclitaxel yield by 37% in the transgenic *Taxus* species cells [115]. To establish a foundational pathway for toxoids, the genome of *T*. *wallichiana* was assembled and has been found to have a genome size of 10.6 Gb. In this genome, a number of genes involved in the biosynthesis of paclitaxel (terpene synthase family TPS002, cytochrome P450 family CYP725, and transferase family TRF004) were found to have undergone significant expansion [114]. These findings suggest that tandem duplication could be the primary mechanism driving the complex paclitaxel biosynthetic pathway. Through tandem duplication, an increasing number of enzymes were created to modify toxoids, ultimately leading to the production of paclitaxel.

There are several key synthetic genes that have been reported in the ginkgolides pathway, including 1-deoxy-D-xylulose-5-phosphate synthase (*DXS*), 1-hydroxy-2-methyl-2-(E)-butenyl-4-diphosphate reductase (*HDR*), geranyl geranyl diphosphate synthase (*GGPPS*), and the levopimaradiene synthase (*LPS*) gene [62,77]. The *GbLPS* was cloned, and it was demonstrated that it could catalyze the synthesis of ginkgolides [116]. Overexpression of *GbHDR2* in *N. tabacum* also elevated the diterpenoid duvatrienediol content at least sixfold [117]. In addition, *CYPs* genes are considered to be linked to ginkgolide biosynthesis, as the suppression of the *GbCYPs* genes can inhibit ginkgolide biosynthesis [118]. In recent research, the first steps of ginkgolide biosynthesis have been revealed through the exploration of gene clusters and co-expression analysis. Five *CYPS* genes (*GbCYP7005C1*, *GbCYP7005C3*, *GbCYP867E3*8, *GbCYP867K1*, and *GbCYP720B31*) were identified in close proximity to the *GbLPS* gene. These genes encode multifunctional enzymes with atypical catalytic activities, resulting in the formation of the tert-butyl group and one of the lactone rings, which are characteristic of all trilactone terpenoids [117]. 

The biosynthetic pathway of cannabinoids differs slightly from that of other terpenoids. Cannabinoid biosynthesis begins with the polyketide pathway’s synthesis of olivetolic acid (OLA) and geranyl pyrophosphate (GPP). Subsequently, an enzyme known as olivetolate geranyltransferase (GOT) facilitates the alkylation of OLA and GPP, resulting in the synthesis of cannabigerolic acid (CBGA) [118,119]. This CBGA can be converted to neutral cannabinoids (THCA, CBDA, and CBCA) through heating, drying, and other non-enzymatic decarboxylation reactions [120]. There is also a variant pathway for cannabinoid production, where divarinolic acid (DA) and GPP generate the cannabinoid precursor, cannabigerovarinic acid (CBGVA), which is then catalyzed by a series of enzymes to eventually form the annabigerovarin (CBGV), Δ9-tetrahydrocannabivarin (THCV), and cannabivarin (CBV). The genes encoding *CsTHCAS*, *CsCBDAS*, and *CsCBCAS* are regarded as key genes in the synthesis process. Among them, *CsTHCAS* and *CsCBDAS* have been shown to promote cannabinoid synthesis in glandular trichosomes [121]. 

## 7. Regulation of Terpenoid Biosynthesis by Transcription Factors

**Figure 4:**
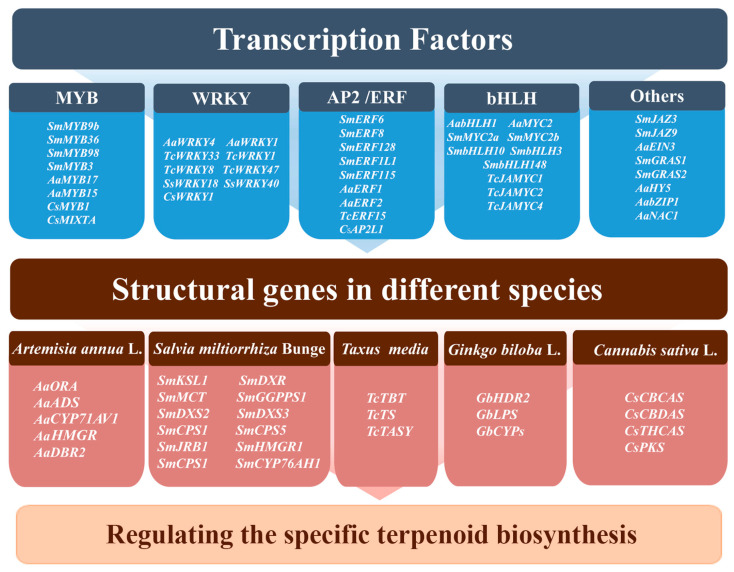
The important TFs and structural genes associated with terpenoid biosynthesis in several important medicinal plants.

Transcription factors (TFs) are DNA-binding proteins that control the transcription of particular genes, thereby controlling their expression within the cell. Several previous studies have identified various TFs that are important in regulating terpenoid biosynthesis (e.g., MYB, WRKY, bHLH, AP2/ERF, and bZIP TFs) (Figure 4). 

MYB serves as a key TF that affects the metabolism of terpenoid compounds. Several MYB TFs were shown to play an important role in terpenoid biosynthesis in medicinal plants. In *S*. *miltiorrhiza*, the transcripts of *SmDXS*, *SmGGPPS*, and *SmKSL1* can be significantly upregulated by SmMYB9b, and an overexpression of *SmMYB97* and *SmMYB98* significantly increases the tanshinone content, indicating that these MYB may play a positive regulatory role in tanshinone biosynthesis [122,123,124]. In *Taxus* species, TmMYB3, a phloem-specific R2R3-MYB, was identified to activate the transcripts of *TmTBT* and *TmTS* to enhance paclitaxel biosynthesis [125]. In *A*. *annua*, AaTAR2 (MYB TF) and AaMYB17 serve as positive regulators for the glandular trichome, and, in turn, increase the level of artemisinin [5,126], while AaMYB15 directly binds to and represses the transcriptional activity of *AaORA*, thus suppressing artemisinin biosynthesis [127]. In *C*. *Sativa*, CsMYB1 can bind *CsCBCAS* and *CsCBDAS* promoters, negatively regulating cannabinoid accumulation [128]. Overexpression of *CsMIXTA* (R2R3-MYB TF) in *N. tabacum* promotes trichoid development, suggesting that it may promote cannabinoid production by increasing the formation of cannabis glandular trichoid [129].

WRKY TFs are specific to plants and have significant functions in defending against pathogens, responding to abiotic factors, and regulating secondary metabolism [130]. Recently, researchers have successfully isolated WRKY genes from various medicinal plants. For example, an overexpression of *AaWRKY1* in *A*. *annua* can significantly activate the expression of artemisinin biosynthetic genes [131]. The overexpression of the *AaWRKY4* gene in *A*. *annua* has the potential to regulate the metabolism of artemisinin, leading to an enhancement in artemisinin biosynthesis [87]. To categorize their impacts on taxol biosynthesis in *Taxus* species, six representative *TcWRKYs* have been selected [74]. Overexpression experiments demonstrated that the six *TcWRKYs* had diverse effects on taxol biosynthesis. Specifically, *TcWRKY8* and *TcWRKY47* significantly and substantially enhanced the expression levels of taxol biosynthesis-related genes [132]. In addition, experimental evidence has shown that *TcWRKY33* has the ability to bind to the w-boxes in the *TcDBAT* promoter, resulting in the promotion of its expression [133]. In *S*. *miltiorrhiza*, SmWRKY1 was found to bind directly to the promoter of *SmDXR* [134], while SmWRKY2 binds to the *SmCPS* promoter to promote tanshinone biosynthesis [135]. In *C*. *sativa*, CsWRKY1 can also influence cannabinoid synthesis by modulating the expression of *CsCBCAS* and *CsCBDAS* [128].

AP2/ERF (APETALA2/ethylene-responsive element binding factors) TFs are a large family in the plant kingdom, and specific AP2/ERF members are essential for terpenoids formation [136,137]. Through experiments like EMSA and transient expression assay, it has been demonstrated that AaERF1, AaERF2, and TAR1 are responsible for regulating sesquiterpenoid biosynthesis in *A*. *annua* through binding to both *AaADS* and *AaCYP71AV1* promoters [138]. In *S*. *miltiorrhiza*, *SmERF6*, *SmERF8*, *SmERF128*, and *SmERF1L1* have also been shown to activate tanshinone biosynthesis [110,137,138,139]. The overexpression of *SmERF1L1* can greatly enhance tanshinone accumulation in transgenic *S*. *miltiorrhiza* hairy roots by extensively upregulating *SmDXR*, which is important for the tanshinone biosynthetic pathway [139]. It was further confirmed that SmERF1L1 can bind directly to the promoter of *SmDXR*, regulating the biosynthesis of tanshinone. In addition, TcERF15 was found to bind and activate *TcTASY*, encoding a key enzyme in *Taxus* species, thereby enhancing paclitaxel biosynthesis [140], as well as CsAP2L1, which can promote the transcript levels of *CsCBCAS* and *CsTHCAS*, suggesting that it promotes cannabinoid biosynthesis [128].

The bHLH TFs have been identified in the regulation of plant growth and development as well as secondary metabolism. Among them, the MYC type in bHLH is the most thoroughly studied transcription factor. In *A*. *annua*, AabHLH1 and AaMYC2 are positive regulatory factors for artemisinin biosynthesis. Overexpression of *AabHLH1* and *AaMYC2* can significantly increase the levels of genes associated with artemisinin biosynthesis, such as *AaADS*, *AaCYP71AV1*, and *AaDBR2* [140,141]. Overexpression of SmbHLH10 and SmbHLH148 in *S*. *miltiorrhiza* can enhance the tanshinone content. Yeast one-hybrid results indicate that SmbHLH10 and SmbHLH148 can directly bind to G-box elements on the promoter of enzyme genes to activate their expression, thereby controlling the tanshinone biosynthesis. Further qRT-PCR analysis of the overexpression lines revealed that *SmDXS*, *SmDXR*, *SmCPS*, and *SmJRB* were greatly induced [142,143]. In contrast, some bHLH TFs suppress terpenoid biosynthesis; SmbHLH3 negatively control tanshinones biosynthesis and downregulates related genes, such as *SmDXR*, *SmDXS3*, *SmHMGR1*, *SmCPS1*, *SmKSL1*, and *SmCYP76AH1* [57]. Similarly, TcJAMYC1, TcJAMYC2, and TcJAMYC4 negatively regulate *Taxus* species paclitaxel biosynthesis [144].

There are several other TFs implicated in terpenoid biosynthesis in medicinal plants. Overexpression of AaNAC1 has been demonstrated to increase the artemisinin levels in *A*. *annua* [43]. AaHY5 and AabZIP1, which belong to the bZIP family, are reported to exert positive control on artemisinin biosynthesis in *A*. *annua* [145,146]. SmJAZ3 and SmJAZ9, which belong to the JAZ family, can inhibit the expression of tanshinone biosynthesis genes [147]. In *S*. *miltiorrhiza*, SmGRAS1 and SmGRAS2, which encode GRAS TFs, can upregulate the transcription of *SmKSL1* to increase the tanshinone levels [112,148]. An increasing number of TFs were identified that are involved in the regulation of terpenoids biosynthesis in medicinal plants. These TFs can act independently or cooperatively to simultaneously regulate terpenoids biosynthesis. However, validating their function and determining their substrate specificity are also significant challenges.

## 8. Conclusions and Perspectives

Over the past decade, advancements in next-generation sequencing techniques, molecular biology techniques, and multi-omics techniques have greatly enhanced our comprehension of the synthesis and regulation of terpenoids in medicinal plants. Environmental factors, structural genes, transcription factors, and transporters have been identified as the pivotal factors influencing the accumulation of terpenoids in these plants. Currently, the development and utilization of key genes or technologies to enhance terpenoid biosynthesis in medicinal plants has become a hot research topic.

However, our knowledge of terpenoids remains incomplete due to the complex and specific nature of their biosynthesis. To further advance our knowledge of terpenoids, future research should focus on several areas: (1) Although several genes upstream of terpenoid biosynthetic pathways have been isolated and cloned, their precise function in the regulation of terpenoid biosynthesis still needs further experimental validation. In addition, little information is available on the downstream terpenoid biosynthetic pathways in medicinal plants, especially regarding *CYP450* family genes. Some specific terpene synthesis pathways still need to be improved. (2) Callus, hairy root, and/or transient overexpression/silencing have been used to assess the functions of terpenoid genes in medicinal plants. However, stable genetic transformation systems are not available for various medicinal plants, such as *S*. *miltiorrhiza*, *G*. *biloba*, and *T*. *media*. Therefore, stable genetic transformation and gene editing systems such as the CRISPR-Cas9 system need to be established to achieve biotechnology breeding. (3) Transporters play an essential role in the transport of terpenoids since the biosynthesis and application sites of terpenoids are different in some medicinal plants. Therefore, it is crucial that we identify and characterize transporters of terpenoids in medicinal plants, which are essential for enhancing the accumulation of terpenoids. (4) Genomics, transcriptomics, and metabolomics analyses have provided insights into terpenoid synthesis in medicinal plants. In the future, more attention should be given to the mechanisms of transcriptional regulation in terpenoid synthesis, as well as the construction of networks of transcriptional and post-transcriptional regulation. Additionally, the influence of epigenetics on terpenoid synthesis should also be considered. (5) The development of biosynthetic techniques, such as the use of yeast and other microorganisms, can facilitate the accumulation of specific terpenoids compared to those produced by plants themselves. However, when multiple terpenoids work together to exert medicinal effects, it becomes challenging to achieve similar outcomes through synthesis. In this regard, plants, capable of producing multiple terpenoids, offer additional advantages. Therefore, future research should focus on enhancing biosynthetic techniques to synthesize multiple terpenoids. These studies will be of great significance for the breeding of medicinal plants with high terpenoid content through biotechnology, as well as the production of terpenoid compounds through bioengineering.
